# Use of Different Food Classification Systems to Assess the Association between Ultra-Processed Food Consumption and Cardiometabolic Health in an Elderly Population with Metabolic Syndrome (PREDIMED-Plus Cohort)

**DOI:** 10.3390/nu13072471

**Published:** 2021-07-20

**Authors:** Celia Martinez-Perez, Rodrigo San-Cristobal, Pilar Guallar-Castillon, Miguel Ángel Martínez-González, Jordi Salas-Salvadó, Dolores Corella, Olga Castañer, Jose Alfredo Martinez, Ángel M. Alonso-Gómez, Julia Wärnberg, Jesús Vioque, Dora Romaguera, José López-Miranda, Ramon Estruch, Francisco J. Tinahones, José Lapetra, Lluis Serra-Majem, Aurora Bueno-Cavanillas, Josep A. Tur, Vicente Martín Sánchez, Xavier Pintó, José J. Gaforio, Pilar Matía-Martín, Josep Vidal, Clotilde Vázquez, Emilio Ros, Maira Bes-Rastrollo, Nancy Babio, Jose V. Sorlí, Camille Lassale, Beatriz Pérez-Sanz, Jessica Vaquero-Luna, María Julia Ajejas Bazán, María Concepción Barceló-Iglesias, Jadwiga Konieczna, Antonio García Ríos, María Rosa Bernal-López, José Manuel Santos-Lozano, Estefanía Toledo, Nerea Becerra-Tomás, Olga Portoles, María Dolores Zomeño, Itziar Abete, Anai Moreno-Rodriguez, Oscar Lecea-Juarez, Stephanie K. Nishi, Júlia Muñoz-Martínez, José M. Ordovás, Lidia Daimiel

**Affiliations:** 1 Nutritional Genomics and Epigenomics Group, Precision Nutrition and Obesity Program, IMDEA Food, CEI UAM + CSIC, 28049 Madrid, Spain; celia.martinez@imdea.org (C.M.-P.); jose.ordovas@tufts.edu (J.M.O.); lidia.daimiel@imdea.org (L.D.); 2 Cardiometabolic Nutrition Group, Precision Nutrition and Cardiometabolic Health Program, IMDEA Food, CEI UAM + CSIC, 28049 Madrid, Spain; jalfmtz@unav.es; 3 Cardiovascular and Nutritional Epidemiology Group, IMDEA Food, CEI UAM + CSIC, 28049 Madrid, Spain; mpilar.guallar@uam.es; 4 Department of Preventive Medicine and Public Health, School of Medicine, Universidad Autónoma de Madrid—IdiPaz Hospital, 28046 Madrid, Spain; 5 Centro de Investigación Biomédica en Red de Epidemiología y Salud Pública (CIBERESP), Instituto de Salud Carlos III, 28029 Madrid, Spain; vioque@umh.es (J.V.); abueno@ugr.es (A.B.-C.); vicente.martin@unileon.es (V.M.S.); jgaforio@ujaen.es (J.J.G.); 6 Welch Center for Prevention, Epidemiology and Clinical Research, Johns Hopkins University, Baltimore, MD 21218, USA; 7 Centro de Investigación Biomédica en Red Fisiopatología de la Obesidad y Nutrición (CIBEROBN), Instituto de Salud Carlos III (ISCIII), 28029 Madrid, Spain; mamartinez@unav.es (M.Á.M.-G.); jordi.salas@urv.cat (J.S.-S.); dolores.corella@uv.es (D.C.); ocastaner@imim.es (O.C.); angelmago13@gmail.com (Á.M.A.-G.); jwarnberg@uma.es (J.W.); mariaadoracion.romaguera@ssib.es (D.R.); jlopezmir@gmail.com (J.L.-M.); restruch@clinic.cat (R.E.); fjtinahones@hotmail.com (F.J.T.); joselapetra543@gmail.com (J.L.); lserra@dcc.ulpgc.es (L.S.-M.); pep.tur@uib.es (J.A.T.); xpinto@bellvitgehospital.cat (X.P.); cvazquezma@gmail.com (C.V.); eros@clinic.cat (E.R.); mbes@unav.es (M.B.-R.); nancy.babio@urv.cat (N.B.); sorli@uv.es (J.V.S.); classale@imim.es (C.L.); luna_jess_@hotmail.com (J.V.-L.); jadzia.konieczna@gmail.com (J.K.); angarios2004@yahoo.es (A.G.R.); josemanuel.santos.lozano@gmail.com (J.M.S.-L.); etoledo@unav.es (E.T.); nerea.becerra@urv.cat (N.B.-T.); olga.portoles@uv.es (O.P.); iabetego@unav.es (I.A.); anai.m.rodriguez@gmail.com (A.M.-R.); 8 Department of Preventive Medicine and Public Health, University of Navarra, IdiSNA, 31009 Pamplona, Spain; 9 Department of Nutrition, Harvard T. H. Chan School of Public Health, Boston, MA 02115, USA; 10 Unitat de Nutrició Humana, Departament de Bioquímica i Biotecnologia, Universitat Rovira i Virgili, 43201 Reus, Spain; stephanie.nishi@urv.cat; 11 Human Nutrition Unit, Institut d’Investigació Sanitària Pere Virgili (IISPV), 43204 Reus, Spain; 12 Department of Preventive Medicine, University of Valencia, 46010 Valencia, Spain; 13 Cardiovascular Risk and Nutrition Research Group (CARIN), Hospital del Mar Medical Research Institute (IMIM), 08003 Barcelona, Spain; mzomeno@imim.es (M.D.Z.); juliamm02@gmail.com (J.M.-M.); 14 Department of Nutrition, Food Sciences and Physiology, University of Navarra, 31009 Pamplona, Spain; beatriz.perez.sanz@navarra.es; 15 Bioaraba Health Research Institute, Osakidetza Basque Health Service, Araba University Hospital, University of the Basque Country UPV/EHU, 01009 Vitoria-Gasteiz, Spain; 16 Department of Nursing, School of Health Sciences, Instituto de Investigación Biomédica de Málaga (IBIMA), University of Málaga, 29016 Málaga, Spain; majejas@ucm.es; 17 Instituto de Investigación Sanitaria y Biomédica de Alicante (ISABIAL-UMH), 03010 Alicante, Spain; 18 Research Group on Nutritional Epidemiology & Cardiovascular Physiopathology (NUTRECOR), Health Research Institute of the Balearic Islands (IdISBa), University Hospital Son Espases (HUSE), 07120 Palma de Mallorca, Spain; 19 Lipids and Atherosclerosis Unit, Department of Internal Medicine, Maimonides Biomedical Research Institute of Cordoba (IMIBIC), Reina Sofia University Hospital, University of Cordoba, 14071 Córdoba, Spain; 20 Department of Internal Medicine, IDIBAPS, Hospital Clinic, University of Barcelona, 08007 Barcelona, Spain; 21 Department of Endocrinology, Instituto de Investigación Biomédica de Málaga (IBIMA), Virgen de la Victoria Hospital, University of Málaga, 29016 Málaga, Spain; 22 Department of Family Medicine, Research Unit, Distrito Sanitario Atención Primaria Sevilla, 41013 Sevilla, Spain; 23 Research Institute of Biomedical and Health Sciences (IUIBS), University of Las Palmas de Gran Canaria and Service of Preventive Medicine, Complejo Hospitalario Universitario Insular Materno Infantil (CHUIMI), Canary Health Service, 35001 Las Palmas de Gran Canaria, Spain; 24 Department of Preventive Medicine and Public Health, University of Granada, 18011 Granada, Spain; 25 Research Group on Community Nutrition & Oxidative Stress, University of Balearic Islands-IUNICS & IDISBA, 07122 Palma de Mallorca, Spain; 26 Institute of Biomedicine (IBIOMED), University of León, 24071 León, Spain; 27 Lipids and Vascular Risk Unit, Internal Medicine, Hospital Universitario de Bellvitge, Hospitalet de Llobregat, 08907 Barcelona, Spain; 28 Departamento de Ciencias de la Salud, Centro de Estudios Avanzados en Olivar y Aceites de Oliva, Universidad de Jaén, 23071 Jaén, Spain; 29 Department of Endocrinology and Nutrition, Instituto de Investigación Sanitaria Hospital Clínico San Carlos (IdISSC), 28040 Madrid, Spain; pilar.matia@gmail.com; 30 Biomedical Research Centre for Diabetes and Metabolic Diseases Network (CIBERDEM), Instituto de Salud Carlos III (ISCIII), 28029 Madrid, Spain; jovidal@clinic.cat; 31 Endocrinology and Nutrition Service, IDIBAPS, Hospital Clinic, University of Barcelona, 08007 Barcelona, Spain; 32 Department of Endocrinology and Nutrition, Hospital Fundación Jiménez Díaz, Instituto de Investigaciones Biomédicas IISFJD, University Autónoma, 28015 Madrid, Spain; 33 Department of Nursing, Faculty of Nursing, Physiotherapy and Podiatry, Universidad Complutense de Madrid, 28040 Madrid, Spain; 34 Centro Salud Cabo Huertas, 03540 Alicante, Spain; conchibarcelo@hotmail.com; 35 Internal Medicine Department, Instituto de Investigación Biomédica de Málaga (IBIMA), Regional University Hospital of Malaga, 29010 Malaga, Spain; rosa.bernal@ibima.eu; 36 School of Health Sciences, Blanquerna-Ramon Llull University, 08001 Barcelona, Spain; 37 Atención Primaria, Osasunbidea, Servicio Navarro de Salud, 31003 Pamplona, Spain; oscar.lecea.juarez@navarra.es; 38 Nutrition and Genomics Laboratory, JM_USDA Human Nutrition Research Center on Aging, Tufts University, Boston, MA 02155, USA

**Keywords:** cardiometabolic risk, classification systems, diet, food processing, IARC, IFIC, NOVA, PREDIMED-Plus, ultra-processed food, UNC

## Abstract

The association between ultra-processed food (UPF) and risk of cardiometabolic disorders is an ongoing concern. Different food processing-based classification systems have originated discrepancies in the conclusions among studies. To test whether the association between UPF consumption and cardiometabolic markers changes with the classification system, we used baseline data from 5636 participants (48.5% female and 51.5% male, mean age 65.1 ± 4.9) of the PREDIMED-Plus (“PREvention with MEDiterranean DIet”) trial. Subjects presented with overweight or obesity and met at least three metabolic syndrome (MetS) criteria. Food consumption was classified using a 143-item food frequency questionnaire according to four food processing-based classifications: NOVA, International Agency for Research on Cancer (IARC), International Food Information Council (IFIC) and University of North Carolina (UNC). Mean changes in nutritional and cardiometabolic markers were assessed according to quintiles of UPF consumption for each system. The association between UPF consumption and cardiometabolic markers was assessed using linear regression analysis. The concordance of the different classifications was assessed with intra-class correlation coefficients (ICC3, overall = 0.51). The highest UPF consumption was obtained with the IARC classification (45.9%) and the lowest with NOVA (7.9%). Subjects with high UPF consumption showed a poor dietary profile. We detected a direct association between UPF consumption and BMI (*p* = 0.001) when using the NOVA system, and with systolic (*p* = 0.018) and diastolic (*p* = 0.042) blood pressure when using the UNC system. Food classification methodologies markedly influenced the association between UPF consumption and cardiometabolic risk markers.

## 1. Introduction

Food processing and its relationship with health is a significant concern due to the impact of this processing on the nutritional food profile [1,2,3,4,5]. This is partly due to the increased consumption of highly industrially processed foods worldwide [6,7,8,9,10,11]. In line with this, the term “ultra-processed food” (UPF) has been introduced in the context of epidemiological studies [12,13,14,15,16,17,18]. Cohort studies have indicated an association between UPF consumption and risk of cardiovascular disease [19,20], type 2 diabetes [21], hypertension [14], irritable bowel syndrome [22], dyslipidemia [23], obesity [13,15,24,25,26,27] and cancer [18]. This association with disease may explain the greater all-cause mortality risk associated with UPF consumption shown by longitudinal studies worldwide [17,28,29,30]. This is relevant since diet in general, and UPF consumption in particular, is a modifiable risk factor for non-communicable diseases; thus, these diseases could be prevented through public health policies that promote healthier food choices and limit UPF consumption [28]. 

Food processing emerged from our ancestors’ needs to preserve food, giving rise to many beneficial qualities. These include the reduction in microbiological risk, creating new products such as bread or fermented foods, fortifying foods, and improving accessibility to nutrients [31,32]. Food processing can also have detrimental effects, as it may involve the loss of essential nutrients, addition of excessive amounts of sugar, salt, fats [8,33,34,35,36], increase in glycemic index [37], additives or even acrylamide [38,39]. This has prompted the negative connotations and perceived poor nutritional profile associated with the term UPF, although this is not always the case [40,41]. Therefore, a clear distinction between UPF and “processed foods” in studies and health reports is critical. Significant controversy has arisen around the effect of food processing on health, given the lack of a standard UPF definition [42]. As a result, studies classify processed foods using different criteria, hence rendering the comparability of their outcomes impractical and somewhat ambiguous. This lack of consistency hinders the identification of UPFs by consumers when following public health guidelines. 

In response to the need to characterize foods according to their processing level, multiple classification systems have been proposed [42]. The most commonly used in nutrition research is produced by NOVA [16,33,43], the International Agency for Research on Cancer (IARC) [11,44], the International Food Information Council (IFIC) [45,46] and the University of North Carolina (UNC) [47] systems. Some are based on the type and level of processing, whereas others focus on formulation and composition criteria. These differences have generated discrepancies in conclusions obtained from studies examining UPF consumption [46,48,49], highlighting the need for a commonly accepted system across studies. 

Here, we aimed to assess the impact of the food classification system on the cross-sectional association between UPF consumption and cardiometabolic health using the same data set. We hypothesized that applying different food processing-based classification systems to a data set would result in different associations between UPF consumption and cardiometabolic markers.

## 2. Materials and Methods

**Figure 1:**
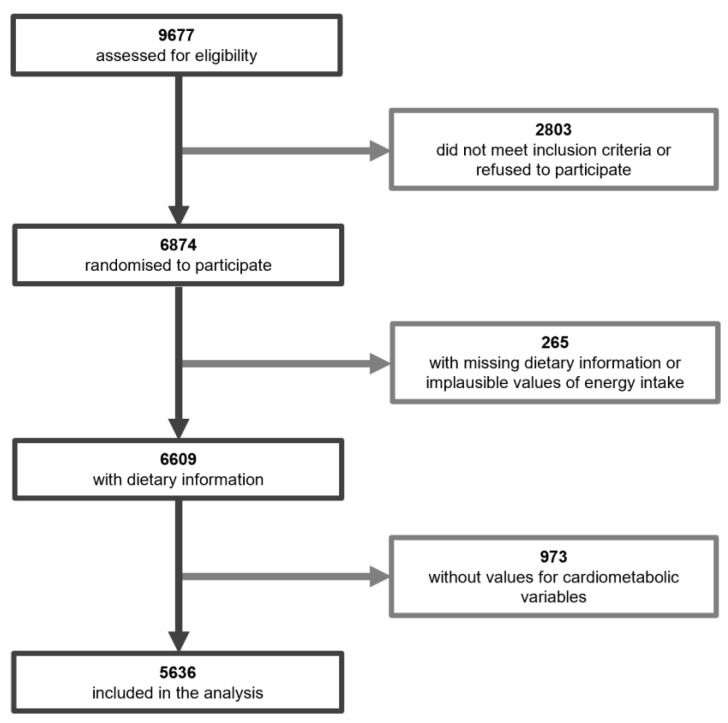
Flowchart of the PREDIMED-Plus participants. Number of subjects shown in bold.

Study population: The PREDIMED-Plus trial (from the Spanish “PREvention with MEDiterranean DIet”) is an ongoing study launched in Spain in 2013 to assess the effect of an intensive lifestyle intervention—energy-restricted Mediterranean diet, increased physical activity and behavioral support—on the incidence of cardiovascular events and weight loss and its long-term maintenance. Details about the cohort have been described previously [50] and the study protocol, including study design and data collection, can be found at the PREDIMED-Plus website (https://www.predimedplus.com/en/, accessed on 20 May 2020). Briefly, the trial involves 6874 participants (48.5% female and 54.5% male) between 55–75 years old (mean age and SD 65.0 ± 4.9) who presented with overweight or obesity (27 ≤ BMI ≤ 40 kg/m^2^) and met at least three criteria for metabolic syndrome (MetS) as previously described [51]. The trial was designed as a 6-year, parallel-group, randomized clinical trial conducted in 23 Spanish study centers. The study protocol was approved according to the ethical standards of the Declaration of Helsinki by the Institutional Review Boards (IRBs) of all participating centers and all participants provided written consent of their participation in the study. This trial was retrospectively registered at the International Standard Randomized Controlled Trial Registry with number 89898870 on 24th July 2014. In the present analysis, baseline data from the PREDIMED-Plus study data set dated 26th June 2020 was used. These data correspond to data at the beginning of the study, before the dietary intervention took place. Participants with missing values for cardiometabolic variables were not included in the analysis. In addition, participants with implausible intakes (<500 or >3500 kcal for females and <800 or >4000 kcal for males) were excluded. The final number of participants included in the analysis was 5636 (48.5% female and 51.5% male, mean age and SD: 65.1 ± 4.9) (Figure 1). This study adhered to the STROBE cross-sectional reporting guidelines [52]. 

Anthropometric and blood measurements: The weight and waist circumference measurements were taken from participants in light clothing with no shoes or accessories, using an electronic calibrated scale and an anthropometric tape, respectively. Waist circumference was measured midway between the lowest rib and the iliac crest. Height measurements were taken using a wall-mounted stadiometer. Body Mass Index (BMI) was calculated as the weight in kilograms divided by the square of height in meters. Blood pressure was measured in triplicate with a sphygmomanometer in a sitting position and after 5 min rest, and the mean systolic and diastolic blood pressure was calculated. Blood samples were collected after overnight fasting by trained nurses at the recruiting centers or primary health care centers associated with the study. Plasma glucose, triglycerides, glycosylated hemoglobin (HbA1c), creatinine, total and HDL cholesterol levels were measured following standard enzymatic methods. LDL cholesterol concentration was calculated using the Friedewald formula [53].

Dietary, nutritional and lifestyle measurements: A semi-quantitative 143-item food frequency questionnaire (FFQ) was used to assess the diet of participants over the last year at baseline, before the dietary intervention. For each food or beverage item, participants were asked the average frequency of consumption of a commonly used portion size for that item (e.g., glass, cup, and slice) over the previous year. Nine options for frequency of consumption are given, ranging from “never or hardly ever” to “more than six times a day.” This FFQ has been repeatedly validated in the Spanish population [54,55,56] and considers variations in dietary patterns among seasons, weekdays and weekends. To estimate the daily consumption for each food or beverage item, the portion size was multiplied by the frequency of consumption and then expressed as grams per day. In the case of nutrient variables, data were estimated from FFQ responses considering all items that contribute to that nutrient intake, using food composition tables explicitly developed for Spanish dietary habits [57,58]. Adherence to an energy-restricted Mediterranean Diet (MedDiet) was assessed using a 17-item questionnaire specially developed and validated for the PREDIMED-Plus trial [59]. Participants were asked about the use or frequency of consumption of traditional Mediterranean food items and scored one point when the answer was in agreement with specific criteria defining energy-restricted MedDiet. Therefore, the higher the score, the better adherence to this diet. The glycemic index (GI) for each participant’s diet was estimated from the calculated glycemic load (GL) for that participant, as the GL multiplied by 100 and divided by the grams of carbohydrates consumed per day. Physical activity and smoking data were collected through the general PREDIMED-Plus questionnaire. Participants were asked about the frequency and intensity of physical activities, and three levels of PA were defined as follows: low (frequent sitting and little walking and/or frequent sitting and moderate sustained efforts), medium (frequent walking with no vigorous efforts), and high (frequent walking and vigorous efforts and/or frequent vigorous efforts). Smoking habits were divided into three groups: never, former or current smoker.

**Figure 2:**
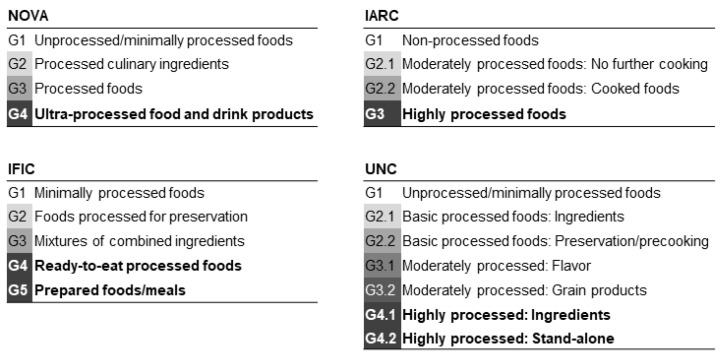
Processing groups defined by each classification system. Darker shades indicate higher processing group. UPF groups as defined in this study shown in bold. IARC: International Agency for Research on Cancer, IFIC: International Food Information Council Foundation, UNC: University of North Carolina.

UPF consumption and food processing-based classification systems: The four systems most described in the literature (NOVA, IARC, IFIC and UNC) were selected based on their relatively broad geographical applicability, the scope of use and availability of food lists. The NOVA system developed by the Public Health Faculty of the University of São Paulo (Brazil) classifies food according to the extent and purpose of their industrial processing [16,33,43]. The IARC system was established in Europe by the International Agency for Research on Cancer (IARC) using the methodology design for the European Prospective Investigation into Cancer and Nutrition (EPIC), and it classifies foods according to their level of physical processing [11,44]. The IFIC system was developed by the International Food Information Council Foundation (IFIC) in the United States (US), and the classifying criteria are based on the increasing complexity of food processing and chemical composition [45,46]. The UNC system was also developed in the US by the University of North Carolina (UNC) and classifies food according to the extent of physicochemical changes produced by food processing [47]. A total of 136 food and beverage items from the FFQ were allocated to processing groups according to food lists and descriptions found in the references cited above for each classification system (Figure 2 and Table S1). The UPF group was defined for each classification system as follows: Group 4 for NOVA, Group 3 for IARC, Groups 4 and 5 for IFIC, and Groups 4.1 and 4.2 for UNC. For each participant, UPF consumption was estimated as the sum of grams per day consumed from foods in the UPF group, divided by the total grams of food consumed per day and multiplied by 100. UPF consumption was calculated according to the items listed in the UPF group of each classification system (Table S1).

The following assumptions were made for particular food items to ensure consistency and avoid bias when applying different classifying criteria. These were based on the foods and cooking habits of the traditional Mediterranean diet in the Spanish population. Therefore, milk was assumed to be processed using ultra-heated temperature (UHT), and milkshakes, soft cheese wedges, custard and ice-cream were assumed to be industrially produced. Meat, fish, eggs and legumes were assumed to be raw or cooked in a basic manner; lettuce was assumed to be consumed raw; cabbage, carrot, peas, pumpkin, zucchini, garlic and onion were assumed to be cooked in a basic manner. When fish was not explicitly described, the same assumptions as for meat were made. Asparagus was assumed to be canned and of the white type. Raisins were assumed to be without added sugar; almonds were assumed to be toasted without salt; walnuts were assumed to be natural with no additions; pistachios were assumed to be salted and other nuts to be salted and/or roasted. Bread was assumed to be freshly baked and not packaged. Muesli included wholegrain cereals with no added sugar; butter was considered as salted, cocoa powder was assumed to contain added sugar, coffee was considered roasted in the form of ground beans (not soluble) and tea was included in the same category as other infusions (e.g., chamomile, green tea).

Statistical analyses. Data analysis was conducted using R programming language [60] in RStudio [61] and with the following statistical packages: “DescTools” [62], “psych” [63], “tableone” [64], “emmeans” [65] and “irr” [66]. Participants were classified according to quintiles of UPF consumption for each classification system (Q1—lowest UPF consumption, Q5—highest UPF consumption). Data shown in tables are presented as “mean (standard deviation, SD)” for continuous variables and as percentages for categorical variables. Statistically significant differences (*p* < 0.05) in cardiometabolic and nutritional variables among UPF quintiles were compared using one-way ANOVA tests. Post hoc analysis was performed using Tukey’s tests to detect pairwise statistically significant differences (*p* < 0.05) between quintiles. Differences between quintiles are expressed in the text as “estimate of the contrast Q1-Q5 ± standard error (SE)”. The association between UPF consumption and cardiometabolic variables (outcome variables) was analyzed with three linear regression models. UPF consumption, expressed as a percentage of total grams of food consumed per day, was divided by five and used as the predictor variable. Thus, regression models show the association between 5% increments in UPF consumption (approximately equivalent to 100 g/day) and cardiometabolic variables. Model 1 included age, gender and recruitment center as cofactors. Model 2 included total energy intake, physical activity level and education level, in addition to cofactors included in model 1. Model 3 included medication for hypertension, cholesterol and type 2 diabetes as cofactors, in addition to those of model 2. Since data used correspond to data before the dietary intervention, the effect of the dietary intervention was not considered for statistical analysis. As sensitive analyses, two alternative estimations of UPF consumption were input in the models: “grams per day of UPF divided by body weight kilogram”, and “calorie-adjusted grams per day of UPF” using the residual method [67]. To assess the concordance between classification systems, the intra-class correlation coefficient (ICC3) was calculated based on a single rating, absolute-agreement, 2-way mixed-effects model, following previously published guidelines [68] and using the “irr” package. Absolute subject agreement among quintiles was calculated as “number of subjects classified in the same quintile in both systems / total number of subjects × 100”. Overall refers to the percentage of individuals classified in the same quintile for any quintile number, whereas Q1 and Q5 refer to the percentage in those specific quintiles.

## 3. Results

### 3.1. UPF Consumption in the PREDIMED-Plus Cohort

The IARC system had the highest number of food items included in the UPF group (60.7% of all FFQ food items), followed by IFIC and UNC (31.1% for both), while NOVA had the lowest number of food items classified as UPF (27.4%) (Figure S1).

**Figure 3:**
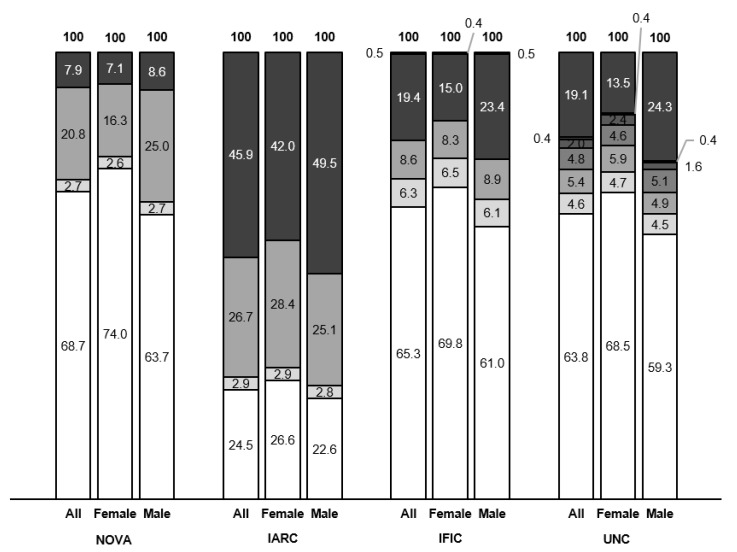
Percentage of consumption by processing group. Numbers indicate mean consumption of foods and beverages in that group over total intake in grams per day. Darker shades indicate higher processing group. UPF groups shown in black at the top.

With 7.9%, NOVA had the lowest percentage of UPF consumption over total consumption in grams per day, whereas IARC, IFIC and UNC showed 45.9%, 20% and 19.7%, respectively (Figure 3). There were differences in UPF consumption between females and males for all systems, with the highest difference shown by UNC, followed by IFIC and IARC and the lowest difference shown by NOVA. In all cases, females showed lower UPF consumption than males.

### 3.2. Nutritional Profile of UPF Consumption Quintiles According to the Classification System

For all classification systems, those subjects in the highest quintile of UPF consumption (Q5) had a higher intake of total energy, saturated fatty acids, simple sugars and sodium and a higher glycemic load compared to those in the lowest quintile (Q1) (Table 1). Likewise, for all systems, a lower intake of protein, fiber and omega-3 fatty acids was observed for subjects in the fifth quintile compared to those in the first quintile. Total fat intake, measured as a percentage of total energy intake, was higher in the highest UPF consumption quintile only with NOVA classification (ΔQ1 − Q5: −1.77 ± 0.28%, Tukey’s *p* < 0.001), while it was lower with IARC (ΔQ1 − Q5: 2.29 ± 0.28%, Tukey’s *p* < 0.001) and UNC (ΔQ1 − Q5: 1.42 ± 0.28%, Tukey’s *p* < 0.001) and did not change with IFIC. The percentage of monounsaturated and polyunsaturated fatty acids intake did not differ across UPF consumption quintiles using NOVA while decreasing with the other classification systems. The percentage of carbohydrates was decreased in Q5 with IFIC (ΔQ1 − Q5: 4.45 ± 0.28%, Tukey’s *p* < 0.001) and UNC (ΔQ1 − Q5: 2.22 ± 0.29%, Tukey’s *p* < 0.001). See Tables S2–S5 for mean values of nutritional markers and ANOVA *p*-values for each classification system.

### 3.3. Association between Cardiometabolic Health Markers and UPF Consumption with Different Classification Systems

We first compared cardiometabolic health markers across UPF consumption quintiles and between Q1 and Q5 consumption quintiles for all systems. There were fewer females, a higher percentage of smokers, and lower MedDiet adherence in the highest quintile of UPF consumption for all classification systems compared to the lowest quintile (see Tables S6–S9). Compared to subjects in the lowest quintile, subjects in the highest quintile were younger and had higher body weight and waist circumference, and lower HDL blood levels. Only in the case of NOVA, those with the highest UPF consumption had significantly higher BMI (ΔQ1 − Q5: −0.72 ± 0.14 kg/m^2^, Tukey’s *p* < 0.001). Levels of HbA1c were only significantly lower in the highest quintile of UPF consumption with IFIC (ΔQ1 − Q5: 0.15 ± 0.04% mmol/mol, Tukey’s *p* < 0.001). Triglycerides levels were significantly higher in Q5 with IARC (ΔQ1 − Q5: −7.07 ± 2.23 mg/dL, Tukey’s *p* = 0.014). Participants in the highest quintile of UPF consumption had lower total cholesterol levels with the NOVA (ΔQ1 − Q5: 4.35 ± 1.57 mg/dL, Tukey’s *p* = 0.045) and UNC classifications (ΔQ1 − Q5: 6.45 ± 1.57 mg/dL, Tukey’s *p* < 0.001). With UNC only, subjects in Q5 had lower LDL cholesterol levels (ΔQ1 − Q5: 4.62 ± 1.38 mg/dL, Tukey’s *p* = 0.007) and higher systolic blood pressure (ΔQ1 − Q5: −2.93 ± 0.711 mmHg, Tukey’s *p* < 0.001) compared to Q1. Subjects with the highest UPF consumption had higher diastolic blood pressure than those with the lowest UPF consumption for all systems except NOVA. 

To analyze the association between UPF consumption and cardiometabolic health markers, we performed linear regression analyses based on a 5% increment in UPF consumption (summarized in Table 2). With the NOVA system, for a 5% increment in UPF consumption, BMI was predicted to increase by 0.11 kg/m^2^, using the fully adjusted model (β = 0.11 kg/m^2^; CI = 0.05, 0.18; *p* = 0.001), whereas an association with higher weight and waist circumference was found with all classification systems (Table 3). A significant direct association between UPF consumption and fasting glucose levels was found with UNC (β = 0.32 mg/dL; CI = 0.02, 0.62; *p* = 0.034), and with Hb1Ac levels in the case of IARC (β = 0.01% mmol/mol; CI = 0, 0.02; *p* = 0.036). A significant inverse association between UPF consumption and HbA1c levels was found with IFIC (*p* < 0.001 and *p* = 0.002 for adjustment models 1 and 2, respectively), although this association was no longer significant after adjustment for medication. No system showed associations with triglycerides or LDL cholesterol levels. We found a significant association between UPF consumption and HDL-cholesterol levels in all classification systems (*p* < 0.001 for all systems), which was positive for all systems but NOVA. A positive association between UPF consumption and total cholesterol levels was found after full adjustment with IARC (β = 0.46 mg/dL; CI = 0.07, 0.84; *p* = 0.021), IFIC (β = 0.88 mg/dL; CI = 0.46, 1.29; *p* < 0.001) and UNC (β = 0.79 mg/dL; CI = 0.38, 1.2; *p* < 0.001). Systolic and diastolic blood pressure showed a direct association with UPF consumption only with UNC (β = 0.25 mmHg; CI = 0.04, 0.46; *p* = 0.018 and β = 0.12 mmHg; CI = 0, 0.23; *p* = 0.042, respectively). The sensitivity analyses revealed very similar associations (data not shown).

### 3.4. Concordance and Subject Agreement between Classification Systems in Quintiles of UPF Consumption

Differences in cardiometabolic markers across quintiles of UPF consumption shown above may be related to subjects being classified to different quintiles for each classification system. Therefore, we calculated ICC3 coefficients to evaluate the concordance of the different classifications to allocate subjects to quintiles of UPF. The ICC3 for the four classifications was 0.51, and all pairwise comparisons with NOVA resulted in lower ICC3 coefficients than the other pairwise comparisons (Table 4). We also conducted a pairwise comparison of the percentage of absolute subject agreement between the quintiles of UPF consumption. Our results showed that IFIC and UNC had the largest percentage of subjects classified in the same quintile (48.6%), and that the percentage of subjects classified to quintile 5 (15.2%) was higher than that of quintile 1 (10.8%). The percentage of subjects in the same quintiles between IARC-IFIC and IARC-UNC was 38.4%. This percentage was very similar for NOVA-IFIC (32.3%) and NOVA-UNC (30%). The highest difference in subjects classified to the same quintiles was observed for the NOVA-IARC comparison (28%), which showed around 7% of subject agreement between quintiles 1 and quintiles 5, the lowest for all pairwise comparisons. 

## 4. Discussion

We analyzed differences in the association between UPF consumption and cardiometabolic markers, as well as in the nutritional profile of subjects with higher UPF consumption compared to subjects with lower UPF consumption, using four food processing-based classification systems on the same dataset. We showed that those with the highest UPF consumption exhibited nutritional markers of a poor-quality diet irrespective of the classification used. In addition, with all classifications, a positive association between UPF consumption and weight and waist circumference was found. Only with NOVA, a positive association between UPF consumption and BMI was detected. Additionally, a positive association with systolic and diastolic blood pressure and fasting glucose levels was found only with UNC, and with HbA1c only using IARC. Marked differences were also detected in subject agreement between quintiles of UPF consumption, with the NOVA-IARC comparison showing the lowest concordance and percentage of subject agreement.

Our results revealed that individuals with the highest UPF consumption (Q5) had a higher intake of energy, simple sugars, saturated fat and sodium, as well as less fiber intake, lower adherence to the MedDiet and a higher glycemic load of the diet than those with the lowest consumption of UPF (Q1), regardless of the classification method (Table 1 and Tables S2–S9). This concurs with previous studies analyzing the nutritional profile of UPFs [33,69,70,71,72]. It is noteworthy that all four systems agreed on results for critical nutrients of which intake has been linked to disease risk, such as sugar [48,73,74], saturated fat [75,76] or sodium [77,78,79]. This consistency in the nutritional profile of subjects with higher UPF consumption supports the claim that food classifications based on food processing are nutritionally relevant, and therefore are useful in epidemiological studies and dietary recommendations [71,80]. Indeed, a recent study revealed that sodium and added sugar were significant predictors of highly processed foods according to the NOVA, UNC and IFIC classifications [70]. 

In support of our initial hypothesis, applying different food processing-based classification systems to the PREDIMED-Plus data set resulted in different associations between UPF consumption and cardiometabolic markers. For example, only with NOVA, the Q5 group showed significantly higher BMI than the Q1 group, which was confirmed by the regression analysis. This concurs with previous studies that have used the NOVA system [13,24,81,82]. Additionally, UPF consumption was directly associated with weight and waist circumference with all classifications, with NOVA showing the highest β value. Notably, many studies use BMI as an obesity-related parameter, and thus the choice of classification system could severely affect the conclusions reached regarding UPF consumption and cardiometabolic risk factors. 

IARC classified the largest number of FFQ food items as UPF (60.7%) as opposed to NOVA (27.4%) (Figure S1). These results agree with a previous study [42] but in contrast to another [70]. This difference may be due to the different sources used to generate the food list utilized for the classification. Since Spanish dietary patterns are part of the MedDiet, the PREDIMED-Plus FFQ considers the cooking habits and distinctive foods of this diet [54,55]. This includes cooking at home and a wide variety of minimally processed foods as main components [83,84]. However, US dietary patterns have been characterized by a higher content of refined grains, sugar, salt and saturated fats [85,86,87,88] and particularly a higher consumption of UPFs compared to other Western countries [6,11,28,47]. Therefore, most foods fall under unprocessed to moderately processed categories when using NOVA on the PREDIMED-Plus FFQ. On the contrary, its use in the context of the US diet, with an already higher presence of UPFs, results in a larger amount of foods allocated into highly processed categories, as in the cited study [70]. This may be the same reason why the UNC system, based on a NOVA adaptation for foods in US supermarkets [47], has more detailed categories and is more permissive with the characteristics of highly processed foods. The specific features of the Western dietary pattern may also underlie the similarities in UPF consumption with IFIC and UNC (Figure 3). These results highlight an important idea: the choice of food classification system should consider the specific characteristics of the diet from which foods are evaluated.

The context in which a food processing-based classification system is applied may also determine which system to use. For instance, the IARC system was framed within cancer research and defines “processing” as any modification to raw food, including even the most basic cooking-at-home methods such as boiling or grilling [11]. This is due to the reported associations of specific cooking methods and processed foods with certain cancers [38,39,89,90]. For instance, processed meat consumption has been reported as a convincing cause of colorectal cancer [91]. Considering this, the orientation of IARC and the EPIC study towards cancer research may result in a more conservative definition of highly industrially processed foods [11], a feature that clearly sets it apart from the other food classification frameworks. Therefore, it seems that the heterogeneity among results obtained with different systems could be partially due to discrepancies in the definition and consequences of food processing, as well as in the under- or overestimation of cooking habits, preservation methods and particular foods (e.g., UHT-milk, olive oil, see Table S1). In addition, remarkable differences in the composition of UPF consumption quintiles, illustrated by the low overall concordance and subject agreement between NOVA and IARC (Table 4), may account for result variability. This analysis demonstrates that differential allocation of food items by each system effectively results in substantial differences in subject grouping, further supporting a strong influence of the food classification system on research outcomes.

Some limitations should be noted when interpreting results from this study. First, this is a cross-sectional analysis and therefore, a causal link between UPF consumption and the analyzed variables cannot be inferred. Second, although the suitability of FFQs for evaluating dietary consumption in large epidemiological studies is undeniable [55], it does present limitations regarding recall bias [56] and nutrient intake estimation [92,93]. The latter is even more pronounced when highly processed foods are considered, as there is a wide variety and they are not present in food composition tables. Therefore, it would be worth considering the inclusion of food processing-criteria when designing FFQs for studies aimed to investigate the role of UPFs on health [80]. Third, assumptions about dietary patterns, cooking methods and food composition and processing were made to ensure consistency when applying the criteria of different systems, but foods may fall into different categories when other dietary patterns are considered. Finally, the PREDIMED-Plus cohort used for our analysis is characterized by the presence of MetS; hence, subjects already have high levels of cardiometabolic risk markers, which may mask potential differences due to UPF consumption. In addition, this is an elderly cohort that probably has lower UPF consumption than younger cohorts.

## 5. Conclusions

In conclusion, the use of different food classifications on the same data set showed that UPF consumption might have a negative impact on the nutritional quality of the diet, whereas its association with cardiometabolic markers differed significantly depending on the system used. With NOVA, high UPF consumption was associated with higher BMI, whereas UNC showed an association with higher glucose levels, systolic and diastolic blood pressure, and IARC with higher HbA1c levels. Additionally, all systems revealed an association with higher weight and waist circumference, and all systems but NOVA with higher total and HDL cholesterol levels. These results support the importance of standardization of criteria to classify processed foods. This will allow epidemiological research to be translated effectively into easily understandable guidelines for the general public. For instance, the development of tools that effectively assess UPF consumption will aid in the identification of food groups of which consumption is excessive or associated with disease marks. Public health recommendations could then include the limitation of consumption of these food groups. In addition, this will help to establish relationships between disease marks and UPF consumption, according to which public health policies could be designed. Therefore, such tools should be available not only for the scientific community with epidemiological purposes, but also for policy makers and clinicians.

## Figures and Tables

**Table 1:** Changes in nutritional variables in quintile 5 compared to quintile 1 of UPF consumption.

	NOVA	IARC	IFIC	UNC
Energy intake (kcal/day)	▲	▲	▲	▲
Protein (g/day)	▲	▲	▲	▲
Protein (% of energy intake)	▼	▼	▼	▼
Total fat (g/day)	▲	▲	▲	▲
Total fat (% of energy intake)	▲	▼	NS	▼
Saturated fat (g/day)	▲	▲	▲	▲
Saturated fat (% of energy intake)	▲	▲	▲	▲
Monounsaturated fat (g/day)	▲	▲	▲	▲
Monounsaturated fat (% of energy intake)	NS	▼	▼	▼
Polyunsaturated (g/day)	▲	▲	▲	▲
Polyunsaturated (% of energy intake)	NS	▼	▼	▼
Carbohydrate (g/day)	▲	▲	▲	▲
Carbohydrate (% of energy intake)	NS	NS	▼	▼
Fiber (g/day)	▼	▼	▼	▼
Simple sugars (g/day)	▲	▲	▲	▲
Sodium (mg/day)	▲	▲	▲	▲
Glycemic Index	▼	▲	▼	▲
Glycemic Load	▲	▲	▲	▲
Omega 3 (g/day)	▼	▼	▼	▼

Arrows indicate direction of change: “▼” decrease, “▲” increase, “NS” not significant change. Statistically significant differences (*p* < 0.05) among quintiles of UPF intake were compared using a one-way ANOVA test. Post hoc analysis was performed using Tukey’s test to detect pairwise statistically significant differences (*p* < 0.05) between quintile 5 and quintile 1.

**Table 2:** Summary of associations between a 5% increment in UPF consumption (% of g/day) and cardiometabolic variables with the fully adjusted linear regression model (model 3).

	NOVA	IARC	IFIC	UNC
Weight (kg)	+	+	+	+
BMI (kg/m^2^)	+	NS	NS	NS
Waist circumference (cm)	+	+	+	+
Glucose (mg/dL)	NS	NS	NS	+
HbA1c (% mmol/mol)	NS	+	NS	NS
Triglycerides (mg/dL)	NS	NS	NS	NS
Total cholesterol (mg/dL)	NS	+	+	+
LDL cholesterol (mg/dL)	NS	NS	NS	NS
HDL cholesterol (mg/dL)	−	+	+	+
Creatinine (mg/dL)	+	NS	NS	NS
Systolic BP (mmHg)	NS	NS	NS	+
Diastolic BP (mmHg)	NS	NS	NS	+

Symbols indicate: “+” positive association, “−” negative association, “NS” not significant association. Model 3 is adjusted for sex, age, recruitment center, total energy intake, physical activity level, education level, medication for blood pressure, cholesterol and diabetes. See Table 3 for values. BMI: Body Mass Index, BP: blood pressure, HbA1c: glycosylated hemoglobin, HDL: high-density lipoprotein, LDL: low-density lipoprotein.

**Table 3:** Associations between cardiometabolic variables and a 5% increment in UPF consumption (% of g/day) by linear regression analysis.

		NOVA			IARC			IFIC			UNC		
	Model	β	CI	*p*	β	CI	*p*	β	CI	*p*	β	CI	*p*
Weight (kg)	1	0.49	(0.27, 0.71)	**<0.001**	0.23	(0.11, 0.36)	**<0.001**	0.25	(0.11, 0.38)	**<0.001**	0.20	(0.06, 0.33)	**0.004**
	2	0.34	(0.12, 0.56)	**0.002**	0.13	(0, 0.26)	0.056	0.17	(0.03, 0.31)	**0.017**	0.12	(−0.02, 0.25)	0.097
	3	0.35	(0.13, 0.57)	**0.002**	0.14	(0.01, 0.27)	**0.033**	0.20	(0.06, 0.34)	**0.005**	0.14	(0, 0.28)	**0.048**
BMI (kg/m^2^)	1	0.14	(0.07, 0.21)	**0.001**	0.04	(0, 0.08)	**0.049**	0.03	(−0.01, 0.08)	0.117	0.04	(0, 0.08)	0.062
	2	0.11	(0.04, 0.18)	**0.001**	0.01	(−0.03, 0.05)	0.501	0.03	(−0.01, 0.07)	0.172	0.03	(−0.01, 0.07)	0.158
	3	0.11	(0.05, 0.18)	**0.001**	0.02	(−0.02, 0.06)	0.397	0.04	(0, 0.08)	0.067	0.04	(−0.01, 0.08)	0.089
Waist circumference (cm)	1	0.34	(0.16, 0.52)	**0.001**	0.23	(0.12, 0.33)	**<0.001**	0.15	(0.04, 0.26)	**0.008**	0.20	(0.09, 0.31)	**<0.001**
	2	0.25	(0.07, 0.42)	**0.006**	0.14	(0.04, 0.25)	**0.008**	0.11	(0, 0.22)	**0.048**	0.16	(0.05, 0.27)	**0.005**
	3	0.26	(0.08, 0.43)	**0.004**	0.15	(0.04, 0.25)	**0.005**	0.15	(0.04, 0.26)	**0.008**	0.18	(0.07, 0.29)	**0.001**
Glucose (mg/dL)	1	−0.43	(−0.99, 0.12)	0.123	0.29	(−0.02, 0.61)	0.07	−0.28	(−0.62, 0.07)	0.115	0.06	(−0.28, 0.39)	0.745
	2	−0.42	(−0.98, 0.14)	0.145	0.33	(0, 0.65)	0.053	−0.23	(−0.59, 0.12)	0.198	0.12	(−0.23, 0.47)	0.497
	3	−0.28	(−0.72, 0.2)	0.254	0.26	(−0.02, 0.54)	0.066	0.16	(−0.14, 0.46)	0.292	0.32	(0.02, 0.62)	0.034
HbA1c (% mmol/mol)	1	0.00	(−0.02, 0.02)	0.907	0.01	(0, 0.02)	**0.045**	−0.02	(−0.03, −0.01)	**<0.001**	−0.01	(−0.02, 0)	**0.02**
	2	0.00	(−0.01,0.02)	0.77	0.01	(0, 0.02)	**0.026**	−0.02	(−0.03, −0.01)	**0.002**	−0.01	(−0.02, 0)	**0.048**
	3	0.01	(−0.01, 0.02)	0.34	0.01	(0, 0.02)	**0.036**	−0.01	(−0.01, 0)	0.31	−0.01	(−0.01, 0)	0.277
Triglycerides (mg/dL)	1	1.05	(−0.01, 2.11)	0.053	0.53	(−0.08, 1.14)	0.089	0.14	(−0.53, 0.8)	0.691	0.48	(−0.16, 1.12)	0.479
	2	0.87	(−0.21, 1.95)	0.114	0.39	(−0.25, 1.02)	0.23	0.03	(−0.66, 0.71)	0.937	0.36	(−0.32, 1.03)	0.299
	3	0.87	(−0.2, 1.95)	0.112	0.36	(−0.28, 0.99)	0.273	0.03	(−0.65, 0.71)	0.931	0.35	(−0.32, 1.02)	0.311
Total cholesterol (mg/dL)	1	−0.33	(−1.04, 0.38)	0.364	0.32	(−0.09, 0.73)	0.122	1.12	(0.68, 1.56)	**<0.001**	0.83	(0.4, 1.26)	**<0.001**
	2	−0.47	(−1.19, 0.25)	0.202	0.27	(−0.15, 0.7)	0.212	1.02	(0.57, 1.48)	**<0.001**	0.77	(0.32, 1.21)	**0.001**
	3	−0.45	(−1.11, 0.21)	0.181	0.46	(0.07, 0.84)	**0.021**	0.88	(0.46, 1.29)	**<0.001**	0.79	(0.38, 1.2)	**<0.001**
LDL cholesterol (mg/dL)	1	−0.19	(−0.82, 0.45)	0.561	−0.04	(−0.41, 0.33)	0.826	0.41	(0.01, 0.81)	**0.043**	0.11	(−0.27, 0.5)	0.565
	2	−0.29	(−0.94, 0.36)	0.383	−0.09	(−0.47, 0.29)	0.63	0.31	(−0.1, 0.72)	0.136	0.02	(−0.38, 0.43)	0.908
	3	−0.25	(−0.83, 0.32)	0.391	0.10	(−0.24, 0.43)	0.578	0.20	(−0.16, 0.57)	0.282	0.07	(−0.29, 0.43)	0.702
HDL cholesterol (mg/dL)	1	−0.35	(−0.56, −0.13)	**0.001**	0.26	(0.14, 0.38)	**<0.001**	0.68	(0.55, 0.81)	**<0.001**	0.62	(0.05, 0.75)	**<0.001**
	2	−0.35	(−0.57, −0.14)	**0.001**	0.29	(0.16, 0.41)	**<0.001**	0.71	(0.57, 0.84)	**<0.001**	0.67	(0.54, 0.81)	**<0.001**
	3	−0.37	(−0.58, −0.15)	**<0.001**	0.29	(0.16, 0.42)	**<0.001**	0.67	(0.54, 0.81)	**<0.001**	0.65	(0.52, 0.78)	**<0.001**
Creatinine (mg/dL)	1	0.01	(0, 0.01)	**<0.001**	0.00	(0, 0)	0.683	0.00	(0, 0)	0.691	0.00	(0, 0)	0.976
	2	0.01	(0, 0.01)	**<0.001**	0.00	(0, 0)	0.424	0.00	(0, 0)	0.715	0.00	(0, 0)	0.641
	3	0.01	(0, 0.01)	**<0.001**	0.00	(0, 0)	0.435	0.00	(0, 0)	0.694	0.00	(0, 0)	0.7
Systolic BP (mmHg)	1	−0.21	(−0.53, 0.12)	0.218	−0.07	(−0.26, 0.12)	0.454	0.03	(−0.17, 0.23)	0.773	0.21	(0.02, 0.41)	0.213
	2	−0.17	(−0.5, 0.16)	0.313	−0.08	(−0.28, 0.11)	0.419	0.05	(−0.16, 0.26)	0.663	0.24	(0.03, 0.44)	**0.045**
	3	−0.17	(−0.5, 0.16)	0.321	−0.08	(−0.27, 0.12)	0.45	0.08	(−0.13, 0.29)	0.482	0.25	(0.04, 0.46)	**0.018**
Diastolic BP (mmHg)	1	0.10	(−0.08, 0.28)	0.274	0.01	(−0.1, 0.11)	0.913	0.10	(−0.02, 0.21)	0.092	0.14	(0.03, 0.25)	**0.013**
	2	0.08	(−0.1, 0.27)	0.378	−0.02	(−0.13, 0.08)	0.661	0.07	(0.04, 0.19)	0.21	0.12	(0, 0.23)	**0.045**
	3	0.08	(−0.1, 0.26)	0.383	−0.01	(−0.12, 0.1)	0.821	0.07	(−0.05, 0.18)	0.252	0.12	(0, 0.23)	**0.042**

Model 1 is adjusted for sex, age and recruitment center. Model 2 is adjusted for sex, age, recruitment center, total energy intake, physical activity level and education level. Model 3 is adjusted for sex, age, recruitment center, total energy intake, physical activity level, education level, medication for blood pressure, cholesterol and diabetes. Significant *p*-values (< 0.05) shown in bold. BMI: Body Mass Index, BP: blood pressure, CI: 95% confidence interval, HbA1c: glycosylated hemoglobin, HDL: high-density lipoprotein, LDL: low-density lipoprotein.

**Table 4:** Subject agreement and concordance between classification systems.

		ICC3	Overall	Q1	Q5
NOVA	IARC	0.32	28.0	7.2	7.8
NOVA	IFIC	0.45	32.3	8.8	8.9
NOVA	UNC	0.38	30.0	8.3	7.4
IARC	IFIC	0.61	38.4	9.8	12.3
IARC	UNC	0.59	38.4	9.4	12.1
IFIC	UNC	0.74	48.6	10.8	15.2

Intra-class correlation coefficient (ICC3) estimates were calculated based on a single rating, absolute-agreement, 2-way mixed-effects model. Percentage of agreement calculated as “number of subjects classified in the same quintile in both systems/total number of subjects × 100”. Overall column indicates percentage of individuals classified in the same quintile for any quintile number, whereas Q1 and Q5 columns indicate the percentage for those specific quintiles. Total number of subjects: *N* = 5636.

## Data Availability

There are restrictions on data availability for the PREDIMED-Plus trial due to the signed consent agreements around data sharing, which only allow access to external researchers for studies following the project purposes. Requestors wishing to access the PREDIMED-Plus trial data used in this study can make a request to the PREDIMED-Plus trial Steering Committee chair: jordi.salas@urv.cat. The request will then be passed to members of the PREDIMED-Plus Steering Committee for deliberation.

## Supplementary Materials

The following are available online at https://www.mdpi.com/article/10.3390/nu13072471/s1, Figure S1: Number of food items from the PREDIMED-Plus food frequency questionnaire by processing group, Table S1: List of food items from the PREDIMED-Plus food frequency questionnaire (FFQ) and their group allocation according to each classification system, Table S2: Mean values of nutritional markers across quintiles of UPF intake according to the NOVA classification, Table S3: Mean values of nutritional markers across quintiles of UPF intake according to the IARC classification, Table S4: Mean values of nutritional markers across quintiles of UPF intake according to the IFIC classification, Table S5: Mean values of nutritional markers across quintiles of UPF intake according to the UNC classification, Table S6: Mean values of cardiometabolic health and sociodemographic markers across quintiles of UPF intake according to the NOVA classification, Table S7: Mean values of cardiometabolic health and sociodemographic markers across quintiles of UPF intake according to the IARC classification, Table S8: Mean values of cardiometabolic health and sociodemographic markers across quintiles of UPF intake according to the IFIC classification, Table S9: Mean values of cardiometabolic health and sociodemographic markers across quintiles of UPF intake according to the UNC classification.
