# Supplementary material for: Use of Different Food Classification Systems to Assess the Association between Ultra-Processed Food Consumption and Cardiometabolic Health in an Elderly Population with Metabolic Syndrome (PREDIMED-Plus Cohort)
Source: Nutrients. 2021 Jul 20;13(7):2471. doi: 10.3390/nu13072471 (PMC8308804; doi:10.3390/nu13072471)
Supplement: Supplementary file 1 [file nutrients-13-02471-s001.zip › nutrients-1259127-supplementary.pdf]

Table S1. List of food items from the PREDIMED-Plus food frequency questionnaire (FFQ) and their group allocation according to each classification system.

FOOD ITEM	NOVA	IARC	IFIC	UNC
**Whole milk**	1	4	1	1
**Semi-skimmed milk**	1	4	1	1
**Skimmed-milk**	1	4	1	1
**Condensed milk**	3	4	3	4
**Cream**	3	4	3	1
**Industrially-produced milkshakes**	4	4	4	4
**Whole-milk yogurt**	1	4	4	3
**Skimmed-milk yogurt**	1	4	4	3
**Flavored Petit Suisse yogurt**	4	4	4	4
**Cottage cheese, curd**	3	4	4	4
**Processed soft cheese wedges**	4	4	3	4
**Cured or semi-cured cheese**	3	4	3	4
**Fresh cheese**	3	4	3	4
**Custard, crème caramel flan, pudding**	4	4	4	7
**Ice-cream**	4	4	4	7
**Eggs**	1	3	1	1
**Skin chicken**	1	3	1	1
**Skinless chicken**	1	3	1	1
**Beef meat**	1	3	1	1
**Pork meat**	1	3	1	1
**Lamb meat**	1	3	1	1
**Rabbit meat**	1	3	1	1
**Liver**	1	3	1	1
**Offal**	1	3	1	1
**Serrano ham**	3	4	4	7
**Sandwich (deli) ham**	4	4	4	7
**Cured cold meats**	4	4	4	7
**Pâté**	4	4	4	6
**Burger**	4	4	5	7
**Bacon, marbling**	3	4	4	4
**White fish**	1	3	1	1
**Blue fish**	1	3	1	1
**Salted cod**	3	4	2	4
**Oysters, mussels and clams**	1	3	1	1
**Calamari, octopus, baby cuttlefish**	1	3	1	1
**Shrimp, prawn, lobster**	1	3	1	1
**Canned fish in water**	3	4	2	4
**Canned fish in oil**	3	4	2	4
**Chard, spinach**	1	3	1	1
**Cabbage, cauliflower, broccoli**	1	3	1	1
**Lettuce, chicory (endive), escarole**	1	1	1	1
**Tomato**	1	1	1	1
**Carrots, pumpkin, squash**	1	3	1	1
**Flat green (Romano) beans**	1	3	1	1
**Zucchini, eggplant, cucumber**	1	3	1	1
**Pepper**	1	3	1	1
**Canned white asparagus**	3	2	2	3
**Gazpacho (cold Spanish tomato soup)**	1	3	1	1
**Other vegetables**	1	3	1	1
**Onions**	1	3	1	1
**Garlic**	1	3	1	1
**Packaged potato crisps/chips**	4	4	4	7

Table S1. Continued

FOOD ITEM	NOVA	IARC	IFIC	UNC
**Homemade French fries/chips**	3	3	1	4
**Boiled/baked potatoes**	1	3	1	1
**Mushrooms**	1	3	1	1
**Oranges, clementines, grapefruit**	1	1	1	1
**Bananas**	1	1	1	1
**Apples, pears**	1	1	1	1
**Strawberries**	1	1	1	1
**Cherries, plums**	1	1	1	1
**Peach, apricot, nectarine**	1	1	1	1
**Watermelon**	1	1	1	1
**Melon**	1	1	1	1
**Kiwi**	1	1	1	1
**Grapes**	1	1	1	1
**Olives**	3	4	2	4
**Canned fruit in juice or syrup**	3	4	2	4
**Raisins**	1	2	2	1
**Toasted almonds**	1	2	1	1
**Salted pistachios**	3	4	1	4
**Walnuts**	1	2	1	1
**Salted/fried nuts**	3	4	1	4
**Lentils**	1	3	1	1
**Dried beans**	1	3	1	1
**Chickpeas**	1	3	1	1
**Peas and beans**	1	3	1	1
**White and sliced bread**	3	4	3	7
**Wholemeal bread**	3	4	3	5
**Breakfast cereals**	4	4	3	7
**Muesli, wholemeal cereals**	1	4	3	1
**White rice**	1	4	1	3
**Wholemeal rice**	1	3	1	1
**Spaghetti, macaroni, Spanish noodles**	1	4	1	3
**Wholemeal pasta**	1	4	1	2
**Pizza**	4	4	5	7
**Refined olive oil**	2	4	2	2
**Extra virgin olive oil**	2	2	2	2
**Olive pomace oil**	2	4	2	2
**Corn (maize) oil**	2	4	2	2
**Sunflower oil**	2	4	2	2
**Soybean oil**	2	4	2	2
**Mixed oil**	2	4	2	2
**Margarine**	4	4	3	6
**Butter**	2	4	3	4
**Lard (animal fat)**	2	4	3	6
**Biscuits**	4	4	4	7
**Wholemeal biscuits**	4	4	4	7
**Chocolate biscuits**	4	4	4	7
**Homemade baking products**	3	3	3	6
**Industrially-produced confectionery**	4	4	4	7
**Donuts**	4	4	4	7
**Muffins, cupcakes**	4	4	4	7
**Industrially-produced cakes**	4	4	4	7
**Churros**	4	4	4	7
**Chocolates**	4	4	4	7
**Soluble cocoa powder**	4	4	4	4
**Nougat**	4	4	4	7

Table S1. Continued

FOOD ITEM	NOVA	IARC	IFIC	UNC
**Marzipan, shortbread biscuits**	4	4	4	6
**Croquettes**	4	4	4	7
**Instant soup**	4	4	3	7
**Mustard**	4	4	3	6
**Mayonnaise**	4	4	3	6
**Tomato sauce, Ketchup**	4	4	3	6
**Table salt**	2	4	2	2
**Jam**	4	4	4	4
**Sugar**	2	4	3	2
**Honey**	2	1	3	1
**Packaged snacks**	4	4	4	7
**Soft drinks**	4	4	4	7
**No-added sugar drinks**	4	4	4	7
**Natural orange juice**	1	1	2	2
**Fruit juice**	1	1	2	2
**Bottled juice**	4	4	4	4
**Decaf coffee**	3	4	1	1
**Coffee**	1	4	1	1
**Tea**	1	3	1	1
**Grape must**	3	4	2	4
**Rosé wine**	3	4	4	7
**Muscatel wine**	3	4	4	7
**Young red wine**	3	4	4	7
**Aged red wine**	3	4	4	7
**White wine**	3	4	4	7
**Spanish sparkling wine (cava)**	3	4	4	7
**Beer**	3	4	4	7
**Liquors, anisette**	4	4	4	7
**Whisky, gin, vodka, cognac**	4	4	4	7

NOVA: [1] Group 1. Unprocessed and minimally processed foods, **[2]** Group 2. Processed culinary ingredients, **[3]** Group 3. Processed foods, **[4]** Group 4. Ultra-processed food and drink products. **International Agency for Research on Cancer (IARC): [1]** Group 1. Non-processed foods, **[2]** Group 2.1. Moderately processed foods: No further cooking, **[3]** Group 2.2. Moderately processed foods: Cooked foods, **[4]** Group 4. Highly processed foods. **International Food Information Council (IFIC): [1]** Group 1. Minimally processed foods, **[2]** Group 2. Foods processed for preservation, **[3]** Group 3. Mixtures of combined ingredients, **[4]** Group 4. Ready-to-eat processed foods, **[5]** Group 5. Prepared foods/meals. **University of North Carolina (UNC): [1]** Group 1. Unprocessed/minimally processed foods, **[2]** Group 2.1. Basic processed foods: Ingredients, **[3]** Group 2.2. Basic processed foods: Preservation/precooking, **[4]** Group 3.1. Moderately processed: Flavor, **[5]** Group 3.2. Moderately processed: Grain products, **[6]** Group 4.1. Highly processed: Ingredients, **[7]** Group 4.2. Highly processed: Stand-alone. For some items such as fruit juices, milkshakes, soups, burgers, ice-cream and pizzas, the PREDIMED-Plus FFQ does not specify whether they are home-made or industrially produced. We assumed they were industrially produced, based on the most common form of consumption in the Spanish population and the Mediterranean diet. In the case of yogurts and whole-grain cereals, the FFQ does not distinguish between plain, sweetened or flavored varieties, so they were assumed to be natural and unsweetened.

Table S2. Mean values of nutritional markers across quintiles of UPF intake according to the NOVA classification.

	NOVA quintiles of UPF intake (% of g/day)					
	**Q1 (lowest)**	**Q2**	**Q3**	**Q4**	**Q5 (highest)**	**p**
n	1128	1127	1127	1127	1127	
UPF consumption (% of g/day)	2.03 (0.74)	4.01 (0.52)	5.97 (0.65)	9.00 (1.17)	18.31 (7.54)	**<0.001**
Energy intake (kcal/day)	2164.60 (507.67)	2292.38 (509.18)	2373.33 (538.54)	2424.60 (541.56)	2498.50 (600.54)	**<0.001***
Protein (g/day)	93.98 (20.95)	96.61 (21.56)	98.68 (22.10)	97.11 (21.51)	98.05 (23.58)	**<0.001***
Protein (% of energy intake)	17.68 (3.05)	17.07 (2.75)	16.86 (2.71)	16.22 (2.47)	15.93 (2.73)	**<0.001***
Total fat (g/day)	92.62 (26.32)	100.06 (26.04)	104.25 (26.91)	108.71 (29.01)	111.78 (31.03)	**<0.001***
Total fat (% of energy intake)	38.55 (7.02)	39.37 (6.34)	39.69 (6.33)	40.44 (6.55)	40.32 (6.36)	**<0.001***
Saturated fat (g/day)	21.54 (6.61)	24.58 (7.18)	26.43 (7.70)	28.06 (8.20)	29.85 (9.84)	**<0.001***
Saturated fat (% of energy intake)	8.96 (1.86)	9.64 (1.77)	10.03 (1.90)	10.41 (1.90)	10.69 (2.11)	**<0.001***
Monounsaturated fat (g/day)	49.53 (16.21)	52.37 (15.45)	53.86 (15.50)	55.82 (16.32)	56.49 (16.69)	**<0.001***
Monounsaturated fat (% of energy intake)	20.62 (5.10)	20.63 (4.60)	20.54 (4.47)	20.83 (4.56)	20.46 (4.34)	0.425
Polyunsaturated (g/day)	16.39 (6.22)	17.39 (6.03)	17.95 (6.42)	18.61 (6.79)	19.20 (7.23)	**<0.001***
Polyunsaturated (% of energy intake)	6.78 (1.94)	6.83 (1.82)	6.80 (1.84)	6.88 (1.84)	6.90 (1.92)	0.537
Carbohydrate (g/day)	219.24 (65.95)	231.52 (66.90)	241.38 (70.99)	245.04 (72.79)	256.66 (78.74)	**<0.001***
Carbohydrate (% of energy intake)	40.47 (7.13)	40.29 (6.70)	40.52 (6.67)	40.28 (7.09)	40.89 (6.69)	0.219
Fiber (g/day)	27.99 (9.33)	26.62 (8.54)	26.30 (8.37)	25.18 (8.40)	23.91 (7.91)	**<0.001***
Simple sugars (g/day)	5.41 (10.66)	6.02 (10.91)	6.47 (10.87)	7.03 (12.60)	8.59 (14.26)	**<0.001***
Sodium (mg/day)	2149.64 (707.04)	2329.08 (721.47)	2456.72 (761.43)	2502.99 (763.87)	2582.58 (840.11)	**<0.001***
Glycemic Index	54.14 (4.91)	54.20 (5.02)	53.95 (4.83)	53.58 (5.55)	53.53 (5.40)	**0.002***
Glycemic Load	120.10 (42.20)	126.87 (43.01)	131.54 (44.53)	132.71 (45.94)	138.74 (48.45)	**<0.001***
Omega-3 (g/day)	0.95 (0.51)	0.91 (0.48)	0.91 (0.47)	0.84 (0.45)	0.82 (0.46)	**<0.001***

Data shown as "mean (standard deviation, SD)". Significant ANOVA p-values (< 0.05) shown in bold. * Significant change in Q5 compared to Q1 (Tukey's p-value < 0.05).

Table S3. Mean values of nutritional markers across quintiles of UPF intake according to the IARC classification.

	IARC quintiles of UPF intake (% of g/day)					
	**Q1 (lowest)**	**Q2**	**Q3**	**Q4**	**Q5 (highest)**	**p**
n	1128	1127	1127	1127	1127	
UPF consumption (% of g/day)	29.58 (5.12)	39.16 (1.96)	45.45 (1.69)	51.95 (2.16)	63.31 (6.23)	
Energy intake (kcal/day)	2121.92 (486.27)	2258.44 (493.28)	2373.94 (534.43)	2437.38 (536.35)	2561.76 (600.21)	**<0.001***
Protein (g/day)	91.39 (20.64)	97.09 (20.96)	98.84 (22.50)	99.07 (21.61)	98.06 (23.34)	**<0.001***
Protein (% of energy intake)	17.46 (2.83)	17.41 (2.64)	16.86 (2.71)	16.50 (2.79)	15.52 (2.65)	**<0.001***
Total fat (g/day)	96.14 (26.97)	101.18 (26.03)	104.89 (28.06)	106.01 (29.44)	109.19 (31.09)	**<0.001***
Total fat (% of energy intake)	40.68 (6.68)	40.40 (6.24)	39.80 (6.39)	39.11 (6.63)	38.39 (6.57)	**<0.001***
Saturated fat (g/day)	22.48 (6.96)	24.83 (7.23)	26.52 (8.11)	27.33 (8.40)	29.30 (9.79)	**<0.001***
Saturated fat (% of energy intake)	9.49 (1.82)	9.89 (1.84)	10.03 (2.02)	10.07 (2.04)	10.26 (2.20)	**<0.001***
Monounsaturated fat (g/day)	51.01 (16.32)	52.87 (15.13)	54.30 (15.90)	54.50 (16.70)	55.38 (16.70)	**<0.001***
Monounsaturated fat (% of energy intake)	21.57 (4.94)	21.16 (4.50)	20.66 (4.50)	20.14 (4.53)	19.54 (4.35)	**<0.001***
Polyunsaturated (g/day)	17.39 (6.38)	17.71 (6.11)	18.11 (6.63)	17.98 (6.61)	18.36 (7.28)	**0.006***
Polyunsaturated (% of energy intake)	7.33 (1.99)	7.02 (1.75)	6.82 (1.79)	6.61 (1.84)	6.41 (1.87)	**<0.001***
Carbohydrate (g/day)	216.21 (63.95)	228.88 (67.04)	242.35 (71.87)	248.60 (72.39)	257.78 (78.07)	**<0.001***
Carbohydrate (% of energy intake)	40.66 (6.81)	40.31 (6.66)	40.66 (6.78)	40.68 (6.96)	40.14 (7.09)	0.203
Fiber (g/day)	29.95 (8.93)	27.68 (8.53)	26.40 (8.17)	24.52 (7.51)	21.43 (7.43)	**<0.001***
Simple sugars (g/day)	3.98 (8.39)	4.78 (9.07)	6.91 (12.10)	7.78 (12.99)	10.09 (15.08)	**<0.001***
Sodium (mg/day)	2051.39 (646.48)	2280.83 (687.74)	2444.22 (724.17)	2536.30 (751.53)	2708.37 (879.06)	**<0.001***
Glycemic Index	52.99 (4.82)	53.62 (4.89)	54.19 (4.93)	54.40 (5.21)	54.21 (5.75)	**<0.001***
Glycemic Load	115.34 (37.95)	123.84 (41.13)	132.62 (45.11)	136.69 (46.37)	141.47 (50.13)	**<0.001***
Omega-3 (g/day)	1.00 (0.50)	0.95 (0.48)	0.90 (0.48)	0.85 (0.45)	0.72 (0.41)	**<0.001***

Data shown as “mean (standard deviation, SD)”. Significant ANOVA p-values (< 0.05) shown in bold. * Significant change in Q5 compared to Q1 (Tukey's p-value < 0.05).

Table S4. Mean values of nutritional markers across quintiles of UPF intake according to the IFIC classification.

	IFIC quintiles of UPF intake (% of g/day)					
	**Q1 (lowest)**	**Q2**	**Q3**	**Q4**	**Q5 (highest)**	**p**
n	1128	1127	1127	1127	1127	
UPF consumption (% of g/day)	7.03 (2.24)	12.51 (1.37)	17.53 (1.58)	24.10 (2.30)	37.88 (8.32)	
Energy intake (kcal/day)	2127.83 (486.77)	2280.63 (512.72)	2328.17 (515.92)	2448.52 (537.93)	2568.29 (599.50)	**<0.001***
Protein (g/day)	93.23 (21.32)	97.80 (21.33)	97.64 (21.63)	98.68 (21.87)	97.08 (23.45)	**<0.001***
Protein (% of energy intake)	17.73 (2.75)	17.38 (2.65)	16.99 (2.65)	16.35 (2.77)	15.31 (2.60)	**<0.001***
Total fat (g/day)	92.67 (26.84)	101.63 (27.15)	103.75 (27.03)	108.46 (28.67)	110.91 (30.26)	**<0.001***
Total fat (% of energy intake)	39.15 (7.08)	40.12 (6.34)	40.19 (6.47)	39.90 (6.29)	39.01 (6.49)	**<0.001**
Saturated fat (g/day)	22.26 (7.05)	25.36 (7.90)	26.18 (7.70)	27.71 (8.49)	28.93 (9.49)	**<0.001***
Saturated fat (% of energy intake)	9.38 (1.92)	9.98 (2.01)	10.11 (1.89)	10.16 (1.98)	10.11 (2.11)	**<0.001***
Monounsaturated fat (g/day)	48.91 (16.15)	52.94 (15.76)	53.57 (15.46)	55.91 (16.19)	56.74 (16.41)	**<0.001***
Monounsaturated fat (% of energy intake)	20.67 (5.08)	20.92 (4.52)	20.82 (4.62)	20.62 (4.40)	20.05 (4.41)	**<0.001***
Polyunsaturated (g/day)	16.52 (6.29)	17.62 (6.26)	17.97 (6.31)	18.52 (6.70)	18.91 (7.25)	**<0.001***
Polyunsaturated (% of energy intake)	6.95 (2.00)	6.93 (1.84)	6.92 (1.82)	6.78 (1.83)	6.60 (1.86)	**<0.001***
Carbohydrate (g/day)	227.44 (68.34)	236.71 (69.93)	237.93 (71.79)	245.04 (72.23)	246.70 (77.42)	**<0.001***
Carbohydrate (% of energy intake)	42.60 (7.13)	41.31 (6.66)	40.61 (6.54)	39.78 (6.38)	38.15 (6.77)	**<0.001***
Fiber (g/day)	28.77 (9.22)	27.36 (8.60)	26.36 (8.44)	25.18 (7.95)	22.32 (7.45)	**<0.001***
Simple sugars (g/day)	4.72 (9.92)	6.25 (11.48)	7.03 (12.09)	7.49 (12.94)	8.04 (12.98)	**<0.001***
Sodium (mg/day)	2154.59 (703.01)	2348.99 (728.31)	2404.92 (755.39)	2514.56 (750.38)	2597.94 (854.90)	**<0.001***
Glycemic Index	54.82 (4.78)	54.16 (4.99)	53.91 (5.01)	53.84 (5.06)	52.67 (5.67)	**<0.001***
Glycemic Load	125.92 (43.53)	129.52 (44.04)	129.60 (45.04)	133.20 (45.29)	131.71 (48.18)	**0.002***
Omega-3 (g/day)	0.90 (0.49)	0.92 (0.48)	0.90 (0.48)	0.87 (0.46)	0.82 (0.45)	**<0.001***

Data shown as “mean (standard deviation, SD)”. Significant ANOVA p-values (< 0.05) shown in bold. * Significant change in Q5 compared to Q1 (Tukey's p-value < 0.05).

Table S5. Mean values of nutritional markers across quintiles of UPF intake according to the UNC classification.

	UNC quintiles of UPF intake (% of g/day)					
	**Q1 (lowest)**	**Q2**	**Q3**	**Q4**	**Q5 (highest)**	**p**
n	1128	1127	1127	1127	1127	
UPF consumption (% of g/day)	5.79 (2.09)	11.59 (1.56)	17.04 (1.69)	24.15 (2.54)	38.88 (8.87)	
Energy intake (kcal/day)	2042.15 (473.92)	2239.65 (491.99)	2364.28 (487.76)	2481.44 (530.53)	2626.00 (583.13)	**<0.001***
Protein (g/day)	93.32 (21.26)	97.59 (21.74)	98.54 (21.50)	98.16 (22.61)	96.83 (22.53)	**<0.001***
Protein (% of energy intake)	18.50 (2.81)	17.60 (2.61)	16.81 (2.45)	15.95 (2.43)	14.89 (2.28)	**<0.001***
Total fat (g/day)	90.60 (27.09)	100.60 (26.83)	104.99 (27.35)	109.75 (27.55)	111.48 (29.76)	**<0.001***
Total fat (% of energy intake)	39.74 (6.98)	40.44 (6.56)	39.95 (6.38)	39.92 (6.24)	38.33 (6.42)	**<0.001***
Saturated fat (g/day)	21.65 (7.20)	25.18 (7.77)	26.60 (8.03)	28.11 (8.21)	28.92 (9.09)	**<0.001***
Saturated fat (% of energy intake)	9.49 (2.00)	10.10 (2.06)	10.07 (1.92)	10.19 (1.95)	9.89 (2.01)	**<0.001***
Monounsaturated fat (g/day)	47.75 (16.05)	52.02 (15.61)	54.30 (15.63)	56.68 (15.49)	57.33 (16.48)	**<0.001***
Monounsaturated fat (% of energy intake)	20.96 (5.03)	20.93 (4.67)	20.72 (4.50)	20.69 (4.42)	19.78 (4.36)	**<0.001***
Polyunsaturated (g/day)	16.24 (6.35)	17.36 (6.23)	18.18 (6.35)	18.78 (6.73)	18.99 (7.05)	**<0.001***
Polyunsaturated (% of energy intake)	7.08 (1.99)	6.95 (1.89)	6.90 (1.89)	6.78 (1.81)	6.48 (1.74)	**<0.001***
Carbohydrate (g/day)	210.67 (61.68)	229.33 (67.11)	243.79 (67.51)	252.28 (75.36)	257.77 (78.54)	**<0.001***
Carbohydrate (% of energy intake)	41.23 (6.93)	40.77 (6.65)	41.11 (6.80)	40.33 (6.75)	39.00 (6.94)	**<0.001***
Fiber (g/day)	30.40 (9.66)	27.38 (8.73)	25.87 (7.63)	24.29 (7.54)	22.04 (6.92)	**<0.001***
Simple sugars (g/day)	4.15 (8.94)	5.75 (11.23)	6.65 (11.76)	8.15 (13.54)	8.83 (13.31)	**<0.001***
Sodium (mg/day)	2023.09 (633.59)	2289.43 (688.50)	2416.17 (717.57)	2576.43 (763.76)	2716.00 (863.13)	**<0.001***
Glycemic Index	52.08 (4.49)	53.31 (4.53)	54.33 (5.09)	55.10 (5.23)	54.58 (5.78)	**<0.001***
Glycemic Load	110.47 (35.98)	122.99 (40.05)	133.34 (41.60)	140.54 (48.03)	142.62 (51.15)	**<0.001***
Omega-3 (g/day)	0.92 (0.50)	0.93 (0.48)	0.89 (0.48)	0.87 (0.46)	0.80 (0.44)	**<0.001***

Data shown as “mean (standard deviation, SD)”. Significant ANOVA p-values (< 0.05) shown in bold. * Significant change in Q5 compared to Q1 (Tukey's p-value < 0.05).

Table S6. Mean values of cardiometabolic and sociodemographic markers across quintiles of UPF intake according to the NOVA classification.

	NOVA quintiles of UPF intake (% of g/day)					
	**Q1 (lowest)**	**Q2**	**Q3**	**Q4**	**Q5 (highest)**	**p**
n	1128	1127	1127	1127	1127	
UPF consumption (% of g/day)	2.03 (0.74)	4.01 (0.52)	5.97 (0.65)	9.00 (1.17)	18.31 (7.54)	
Female sex (%)	649 (57.5)	577 (51.2)	543 (48.2)	513 (45.5)	454 (40.3)	**<0.001***
Age (years)	66.27 (4.60)	65.50 (4.82)	64.95 (4.75)	64.57 (4.89)	64.03 (5.21)	**<0.001***
Smoking status (%)						**<0.001**
Current smoker	113 (10.0)	126 (11.2)	124 (11.1)	136 (12.2)	166 (14.8)	
Former smoker	465 (41.3)	468 (41.7)	510 (45.5)	516 (46.2)	495 (44.0)	
Never smoker	547 (48.6)	528 (47.1)	488 (43.5)	466 (41.7)	463 (41.2)	
Type II diabetes (%)	334 (29.7)	313 (27.9)	301 (26.8)	295 (26.2)	296 (26.3)	0.316
Hypertension (%)	924 (82.5)	959 (85.7)	958 (85.6)	925 (82.8)	947 (84.3)	0.106
Physical activity (%)						0.2
Low	531 (50.0)	561 (52.8)	559 (52.0)	585 (54.2)	618 (56.5)	
Medium	469 (44.1)	437 (41.1)	451 (41.9)	437 (40.5)	416 (38.0)	
High	63 (5.9)	64 (6.0)	66 (6.1)	57 (5.3)	60 (5.5)	
MedDiet adherence (p17)	9.75 (2.55)	8.93 (2.58)	8.55 (2.44)	8.07 (2.45)	7.47 (2.59)	**<0.001***
Weight (kg)	83.76 (11.77)	85.76 (12.80)	86.76 (12.81)	87.52 (12.97)	88.69 (13.40)	**<0.001***
BMI (kg/m2)	32.12 (3.27)	32.57 (3.41)	32.61 (3.42)	32.59 (3.52)	32.84 (3.53)	**<0.001***
Waist circumference (cm)	105.77 (8.99)	107.10 (9.52)	107.58 (9.30)	108.32 (9.95)	108.99 (9.82)	**<0.001***
Glucose (mg/dL)	113.05 (27.32)	114.14 (28.94)	113.00 (27.36)	113.19 (27.57)	112.40 (27.72)	0.68
HbA1c (% mmol/mol)	6.10 (0.80)	6.11 (0.83)	6.08 (0.79)	6.10 (0.90)	6.10 (0.86)	0.922
Triglycerides (mg/dL)	137.61 (52.35)	140.89 (53.85)	143.24 (52.23)	143.03 (53.66)	143.29 (53.24)	**0.047**
Total cholesterol (mg/dL)	197.25 (37.31)	195.87 (37.22)	197.80 (37.34)	195.77 (36.54)	192.90 (38.04)	**0.02***
LDL cholesterol (mg/dL)	120.07 (33.49)	119.14 (32.52)	120.90 (32.41)	119.55 (32.37)	117.43 (33.08)	0.135
HDL cholesterol (mg/dL)	49.65 (11.93)	48.55 (10.90)	48.24 (11.38)	47.63 (11.97)	46.83 (11.72)	**<0.001***
Creatinine (mg/dL)	0.82 (0.19)	0.83 (0.21)	0.83 (0.19)	0.84 (0.21)	0.86 (0.21)	**<0.001***
Systolic BP (mmHg)	140.10 (17.39)	140.21 (16.89)	140.06 (16.48)	138.82 (17.23)	138.48 (16.48)	**0.033**
Diastolic BP (mmHg)	80.63 (9.55)	80.50 (9.75)	80.88 (10.07)	80.95 (9.78)	80.93 (10.40)	0.759

Data shown as "mean (standard deviation, SD)" for continuous variables and as "number of subjects (percentage)" for categorical variables. Significant ANOVA p-values (< 0.05) shown in bold. * Significant change in Q5 compared to Q1 (Tukey's p-value < 0.05). BMI: Body Mass Index, BP: blood pressure, HbA1c: glycosylated hemoglobin, HDL: high-density lipoprotein, LDL: low-density lipoprotein, MedDiet: Mediterranean diet.

Table S7. Mean values of cardiometabolic and sociodemographic markers across quintiles of UPF intake according to the IARC classification.

	IARC quintiles of UPF intake (% of g/day)					
	**Q1 (lowest)**	**Q2**	**Q3**	**Q4**	**Q5 (highest)**	**p**
n	1128	1127	1127	1127	1127	
UPF consumption (% of g/day)	29.58 (5.12)	39.16 (1.96)	45.45 (1.69)	51.95 (2.16)	63.31 (6.23)	
Female sex (%)	771 (68.4)	653 (57.9)	571 (50.7)	476 (42.2)	265 (23.5)	**<0.001***
Age (years)	65.99 (4.49)	65.77 (4.88)	65.40 (4.72)	64.63 (4.95)	63.52 (5.12)	**<0.001***
Smoking status (%)						**<0.001**
Current smoker	93 (8.3)	115 (10.2)	127 (11.3)	136 (12.2)	194 (17.3)	
Former smoker	404 (35.8)	434 (38.6)	495 (44.2)	540 (48.3)	581 (51.7)	
Never smoker	630 (55.9)	574 (51.1)	497 (44.4)	443 (39.6)	348 (31.0)	
Type II diabetes (%)	313 (27.8)	308 (27.4)	320 (28.5)	301 (26.8)	297 (26.4)	0.823
Hypertension (%)	947 (84.2)	949 (84.8)	932 (83.1)	948 (85.1)	937 (83.7)	0.715
Physical activity (%)						**<0.001**
Low	552 (50.7)	546 (50.8)	587 (54.5)	559 (52.3)	610 (57.2)	
Medium	488 (44.9)	478 (44.5)	424 (39.4)	439 (41.1)	381 (35.7)	
High	48 (4.4)	50 (4.7)	66 (6.1)	70 (6.6)	76 (7.1)	
MedDiet adherence (p17)	9.64 (2.47)	9.12 (2.53)	8.58 (2.58)	8.18 (2.42)	7.26 (2.54)	**<0.001***
Weight (kg)	83.36 (12.54)	84.48 (12.15)	86.24 (12.60)	87.26 (12.70)	91.15 (12.95)	**<0.001***
BMI (kg/m2)	32.59 (3.50)	32.46 (3.44)	32.63 (3.49)	32.41 (3.36)	32.65 (3.40)	0.35
Waist circumference (cm)	105.74 (9.74)	106.43 (9.41)	107.51 (9.53)	108.15 (9.57)	109.93 (9.10)	**<0.001***
Glucose (mg/dL)	112.12 (26.77)	111.18 (25.20)	113.57 (29.52)	114.25 (28.23)	114.67 (28.87)	**0.013**
HbA1c (% mmol/mol)	6.09 (0.79)	6.10 (0.80)	6.09 (0.86)	6.10 (0.83)	6.11 (0.90)	0.949
Triglycerides (mg/dL)	139.55 (51.35)	141.63 (53.22)	139.36 (52.24)	140.90 (53.12)	146.62 (55.23)	**0.007***
Total cholesterol (mg/dL)	196.97 (37.92)	199.10 (37.63)	195.68 (36.27)	194.12 (36.52)	193.70 (38.02)	**0.003**
LDL cholesterol (mg/dL)	120.03 (34.18)	121.69 (32.87)	119.36 (31.95)	117.71 (31.56)	118.30 (33.20)	**0.037**
HDL cholesterol (mg/dL)	49.03 (11.51)	49.09 (11.73)	48.46 (11.61)	48.25 (11.77)	46.08 (11.24)	**<0.001***
Creatinine (mg/dL)	0.81 (0.20)	0.82 (0.21)	0.83 (0.20)	0.85 (0.20)	0.88 (0.20)	**<0.001***
Systolic BP (mmHg)	139.00 (17.45)	140.31 (17.37)	139.21 (16.84)	140.03 (16.24)	139.13 (16.61)	0.239
Diastolic BP (mmHg)	79.82 (9.66)	80.24 (9.77)	80.73 (9.89)	81.55 (9.94)	81.55 (10.20)	**<0.001***

Data shown as “mean (standard deviation, SD)” for continuous variables and as “number of subjects (percentage)” for categorical variables. Significant ANOVA p-values (< 0.05) shown in bold. * Significant change in Q5 compared to Q1 (Tukey's p-value < 0.05). BMI: Body Mass Index, BP: blood pressure, HbA1c: glycosylated hemoglobin, HDL: high-density lipoprotein, LDL: low-density lipoprotein, MedDiet: Mediterranean diet.

Table S8. Mean values of cardiometabolic and sociodemographic markers across quintiles of UPF intake according to the IFIC classification.

	IFIC quintiles of UPF intake (% of g/day)					
	**Q1 (lowest)**	**Q2**	**Q3**	**Q4**	**Q5 (highest)**	**p**
n	1128	1127	1127	1127	1127	
UPF consumption (% of g/day)	7.03 (2.24)	12.51 (1.37)	17.53 (1.58)	24.10 (2.30)	37.88 (8.32)	
Female sex (%)	817 (72.4)	727 (64.5)	554 (49.2)	415 (36.8)	223 (19.8)	**<0.001***
Age (years)	66.31 (4.37)	65.83 (4.70)	65.09 (4.99)	64.54 (5.03)	63.55 (5.00)	**<0.001***
Smoking status (%)						**<0.001**
Current smoker	102 (9.1)	108 (9.6)	139 (12.4)	136 (12.1)	180 (16.1)	
Former smoker	352 (31.3)	412 (36.7)	490 (43.6)	563 (50.3)	637 (56.8)	
Never smoker	671 (59.6)	602 (53.7)	494 (44.0)	421 (37.6)	304 (27.1)	
Type II diabetes (%)	395 (35.1)	312 (27.7)	276 (24.6)	276 (24.6)	280 (24.9)	**<0.001**
Hypertension (%)	962 (85.8)	933 (83.2)	944 (84.4)	934 (83.3)	940 (84.2)	0.449
Physical activity (%)						**0.004**
Low	579 (53.7)	607 (56.3)	539 (50.6)	554 (51.2)	575 (53.7)	
Medium	450 (41.7)	424 (39.3)	468 (43.9)	445 (41.2)	423 (39.5)	
High	49 (4.5)	47 (4.4)	59 (5.5)	82 (7.6)	73 (6.8)	
MedDiet adherence (p17)	9.28 (2.53)	8.77 (2.53)	8.65 (2.57)	8.37 (2.62)	7.70 (2.68)	**<0.001***
Weight (kg)	82.20 (11.74)	84.36 (12.14)	86.23 (12.82)	88.40 (12.90)	91.30 (12.76)	**<0.001***
BMI (kg/m2)	32.48 (3.48)	32.65 (3.47)	32.52 (3.48)	32.52 (3.41)	32.57 (3.36)	0.81
Waist circumference (cm)	105.18 (9.60)	106.76 (9.42)	107.17 (9.70)	108.58 (9.29)	110.08 (9.17)	**<0.001***
Glucose (mg/dL)	115.24 (29.87)	111.71 (26.07)	112.11 (27.69)	113.24 (27.86)	113.49 (27.20)	**0.024**
HbA1c (% mmol/mol)	6.21 (0.90)	6.10 (0.77)	6.08 (0.80)	6.05 (0.84)	6.05 (0.86)	**<0.001***
Triglycerides (mg/dL)	140.83 (51.94)	142.60 (52.10)	140.34 (53.46)	140.97 (54.11)	143.31 (53.87)	0.627
Total cholesterol (mg/dL)	196.52 (37.22)	198.29 (37.40)	195.42 (36.28)	194.90 (36.76)	194.44 (38.83)	0.106
LDL cholesterol (mg/dL)	119.11 (33.18)	121.35 (33.13)	119.38 (31.96)	118.91 (31.81)	118.34 (33.78)	**0.238**
HDL cholesterol (mg/dL)	49.24 (11.26)	48.42 (11.20)	47.99 (11.93)	47.81 (11.96)	47.45 (11.67)	**0.003***
Creatinine (mg/dL)	0.79 (0.19)	0.81 (0.19)	0.84 (0.21)	0.86 (0.21)	0.88 (0.20)	**<0.001***
Systolic BP (mmHg)	139.61 (17.70)	139.38 (16.93)	138.86 (16.68)	139.27 (16.68)	140.55 (16.49)	0.184
Diastolic BP (mmHg)	79.56 (9.40)	80.09 (9.90)	80.98 (9.51)	81.19 (10.26)	82.08 (10.28)	**<0.001***

Data shown as "mean (standard deviation, SD)" for continuous variables and as "number of subjects (percentage)" for categorical variables. Significant ANOVA p-values (< 0.05) shown in bold. * Significant change in Q5 compared to Q1 (Tukey's p-value < 0.05). BMI: Body Mass Index, BP: blood pressure, HbA1c: glycosylated hemoglobin, HDL: high-density lipoprotein, LDL: low-density lipoprotein, MedDiet: Mediterranean diet.

Table S9. Mean values of cardiometabolic and sociodemographic markers across quintiles of UPF intake according to the UNC classification.

	UNC quintiles of UPF intake (% of g/day)					
	**Q1 (lowest)**	**Q2**	**Q3**	**Q4**	**Q5 (highest)**	**p**
n	1128	1127	1127	1127	1127	
UPF consumption (% of g/day)	5.79 (2.09)	11.59 (1.56)	17.04 (1.69)	24.15 (2.54)	38.88 (8.87)	
Female sex (%)	891 (79.0)	750 (66.5)	547 (48.5)	360 (31.9)	188 (16.7)	**<0.001***
Age (years)	66.29 (4.43)	65.95 (4.62)	65.29 (4.83)	64.33 (5.15)	63.46 (4.96)	**<0.001***
Smoking status (%)						**<0.001**
Current smoker	86 (7.6)	101 (9.0)	125 (11.2)	160 (14.3)	193 (17.2)	
Former smoker	332 (29.5)	402 (35.8)	494 (44.1)	584 (52.0)	642 (57.3)	
Never smoker	707 (62.8)	621 (55.2)	501 (44.7)	378 (33.7)	285 (25.4)	
Type II diabetes (%)	331 (29.4)	320 (28.5)	315 (28.0)	298 (26.5)	275 (24.5)	0.081
Hypertension (%)	945 (84.1)	921 (82.5)	959 (85.5)	949 (84.7)	939 (84.2)	0.369
Physical activity (%)						**<0.001**
Low	558 (51.3)	597 (55.8)	584 (54.3)	584 (54.0)	531 (50.1)	
Medium	486 (44.7)	427 (39.9)	421 (39.1)	432 (40.0)	444 (41.9)	
High	44 (4.0)	45 (4.2)	71 (6.6)	65 (6.0)	85 (8.0)	
MedDiet adherence (p17)	10.07 (2.42)	8.89 (2.37)	8.38 (2.51)	7.98 (2.54)	7.46 (2.57)	**<0.001***
Weight (kg)	82.18 (11.88)	83.95 (12.24)	86.02 (12.46)	88.89 (12.92)	91.45 (12.64)	**<0.001***
BMI (kg/m2)	32.63 (3.49)	32.74 (3.51)	32.42 (3.43)	32.45 (3.36)	32.49 (3.39)	0.135
Waist circumference (cm)	105.19 (9.75)	106.31 (9.53)	107.24 (9.21)	109.02 (9.43)	109.99 (9.17)	**<0.001***
Glucose (mg/dL)	112.35 (26.43)	112.66 (28.43)	113.38 (29.42)	113.67 (27.53)	113.72 (27.03)	0.691
HbA1c (% mmol/mol)	6.14 (0.81)	6.13 (0.82)	6.09 (0.84)	6.08 (0.87)	6.05 (0.84)	**0.044**
Triglycerides (mg/dL)	139.91 (52.81)	141.82 (52.62)	139.95 (52.04)	141.47 (53.44)	144.90 (54.45)	0.158
Total cholesterol (mg/dL)	200.26 (37.22)	197.55 (37.69)	194.36 (36.55)	193.61 (36.54)	193.81 (38.18)	**<0.001***
LDL cholesterol (mg/dL)	122.37 (33.32)	120.38 (33.46)	118.25 (32.05)	118.35 (31.96)	117.75 (32.94)	**0.003***
HDL cholesterol (mg/dL)	49.91 (11.35)	48.80 (11.43)	48.12 (11.67)	46.98 (11.90)	47.10 (11.49)	**<0.001***
Creatinine (mg/dL)	0.79 (0.19)	0.79 (0.19)	0.85 (0.22)	0.87 (0.21)	0.88 (0.19)	**<0.001***
Systolic BP (mmHg)	138.02 (16.84)	138.50 (17.25)	140.09 (16.86)	140.13 (16.89)	140.94 (16.55)	**<0.001***
Diastolic BP (mmHg)	79.04 (9.57)	79.97 (9.66)	80.31 (9.66)	82.22 (10.14)	82.36 (10.10)	**<0.001***

Data shown as “mean (standard deviation, SD)” for continuous variables and as “number of subjects (percentage)” for categorical variables. Significant ANOVA p-values (< 0.05) shown in bold. * Significant change in Q5 compared to Q1 (Tukey's p-value < 0.05). BMI: Body Mass Index, BP: blood pressure, HbA1c: glycosylated hemoglobin, HDL: high-density lipoprotein, LDL: low-density lipoprotein, MedDiet: Mediterranean diet.

Supplementary Material

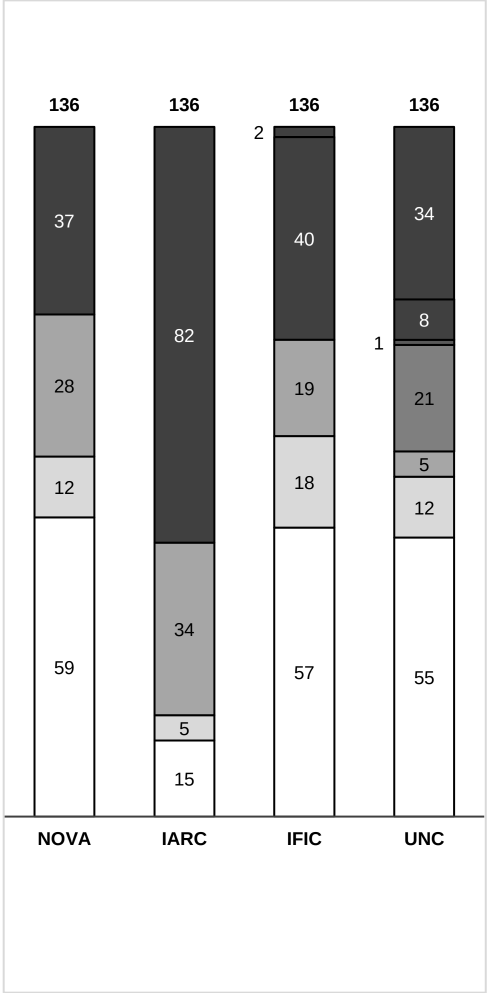

Figure S1. Number of food items from the PREDIMED-Plus food frequency questionnaire by processing group. Darker shades indicate higher processing group.
